# Population Transformer: Learning Population-Level Representations of Neural Activity

**Published:** 2024-10-09

**Authors:** Geeling Chau, Christopher Wang, Sabera Talukder, Vighnesh Subramaniam, Saraswati Soedarmadji, Yisong Yue, Boris Katz, Andrei Barbu

## Abstract

We present a self-supervised framework that learns population-level codes for arbitrary ensembles of neural recordings at scal. We address two key challenges in scaling models with neural time-series data: sparse and variable electrode distribution across subjects and datasets. The Population Transformer (PopT) stacks on top of pretrained representations and enhances downstream decoding by enabling learned aggregation of multiple spatially-sparse data channels. The pretrained PopT lowers the amount of data required for downstream decoding experiments, while increasing accuracy, even on held-out subjects and tasks. Compared to end-to-end methods, this approach is computationally lightweight and more interpretable, while still retaining competitive performance. We further show how our framework is generalizable to multiple time-series embeddings and neural data modalities. Beyond decoding, we interpret the pretrained PopT and fine-tuned models to show how they can be used to extract neuroscience insights from massive amounts of data. We release our code as well as a pretrained PopT to enable off-the-shelf improvements in multi-channel intracranial data decoding and interpretability.

## 1 Introduction

Building effective representations of neural data is an important tool in enabling neuroscience research. We are particularly interested in modeling neural activity recorded from spatially varying electrode layouts, such as intracranial recordings, which use invasive probes placed within the brain to provide high temporal resolution recordings of local neural activity (Parvizi & Kastner, 2018; Herff et al., 2020). But because of dispersed electrode placement within the brain volume, intracranial recordings (iEEG) suffer from data sparsity, and there is significant variability in probe placement across subjects (Parvizi & Kastner, 2018; Herff et al., 2020), leading to high variability in the semantics of the input channels. While these difficulties are particularly relevant for iEEG, they also exist for other neural data modalities such as non-invasive EEG due to variation in electrode layouts. Furthermore, annotated training data is often scarce, since it is limited by patient availability and labor-intensive labeling. This is a problem for many existing approaches which rely on supervised learning (Faezi et al., 2021; Herff et al., 2020; Martin et al., 2018; Metzger et al., 2023; Willett et al., 2023).

To improve decoding data-efficiency, self-supervised pretraining on unannotated data can be employed to first learn generic representations of the recordings, independent of the downstream decoding task that requires task labels. This means that the model does not have to use valuable annotated samples to learn how to do feature extraction before it can do classification, improving the reach of neuroscientific research. In this paper, we are interested in developing generic representations of multi-channel intracranial recordings that enable efficient adaptation to a wide range of downstream decoding tasks. Prior work has shown how to pretrain subject-specific (Le & Shlizerman, 2022) or channel-specific (Wang et al., 2022) models of intracranial data, but such techniques ignore inter-channel relationships or commonalities that might exist across subjects. The most general approach would be to pretrain using data from multiple datasets, but would require tackling the aforementioned challenges of sparse electrode coverage and variable electrode placement between subjects.

We propose Population Transformer (PopT), a self-supervised pretraining approach that learns subject-generic representations of arbitrary electrode ensembles. Transformers offer the flexibility to learn aggregate information across channel configurations, but massive data is needed to train the attention weights (Devlin et al., 2019). During pretraining, we train on large amounts of unannotated data and simultaneously optimize both a channel-level and ensemble-level objective. This requires the model to (1) build subject-generic representations of channel ensembles and (2) meaningfully distinguish temporal relationships between different ensembles of channels.

Our PopT approach is modular, and builds on top of powerful single-channel temporal embeddings, which provides two key advantages. First, by separating the single-channel embedding and multi-channel-aggregation into different modules, we make our approach agnostic to the specific type of temporal embedding used, leaving room for future independent improvements along either the temporal or spatial dimension (an approach that has been validated in video modeling (Arnab et al., 2021)). Second, by taking advantage of learned channel embeddings, PopT training is computationally lightweight compared to their end-to-end counterparts (Appendix B) and baseline aggregation approaches (Figure 4), allowing for adoption in lower compute resource environments.

Empirically, we find that our pretrained PopT outperforms commonly used aggregation approaches (Ghosal & Abbasi-Asl, 2021), and is competitive with end-to-end trained methods (Zhang et al., 2024; Yang et al., 2024; You et al., 2019). Moreover, we find that these benefits hold even for subjects not seen during pretraining, indicating its usefulness for new subject decoding. We also show that the pretrained PopT weights themselves reveal interpretable patterns for neuroscientific study. Finally, we demonstrate that our proposed framework is agnostic to the underlying temporal encoder further allowing it to adapt to other neural recording modalities.

Our main contributions are:

1. a generic self-supervised learning framework, Population Transformer (PopT), that learns joint representations of arbitrary channel ensembles across neural datasets,
2. a demonstration that pretraining systematically improves ensemble representations for downstream decoding even for held-out subjects,
3. a new method for brain region connectivity analysis and functional brain region identification based on the pretrained and fine-tuned PopT weights,
4. a trained and usable off-the-shelf model that computes population-level representations of high temporal resolution intracranial neural recordings.

## 2 Related Work

### Self-supervised learning on neural data

Channel independent pretrained models are a popular approach for neural spiking data (Liu et al., 2022), intracranial brain data (Wang et al., 2022; Talukder & Gkioxari, 2023), and general time-series (Talukder et al., 2024). Additionally, in fixed-channel neural datasets, approaches exist for EEG (Chien et al., 2022; Kostas et al., 2021; Yi et al., 2023), fMRI (Thomas et al., 2022; Kan et al., 2022; Ortega Caro et al., 2023), and calcium imaging (Antoniades et al., 2023) datasets. However, these approaches do not learn population-level interactions across datasets with different recording layouts, either due to a single-channel focus or the assumption that the channel layout is fixed. Several works pretrain spatial and temporal dimensions across datasets with variable inputs (Zhang et al., 2024; Yang et al., 2024; Jiang et al., 2024; Ye et al., 2024; Cai et al., 2023), but most simultaneously learn the temporal embeddings with the spatial modeling, which make them challenging to interpret and computationally expensive to train, especially for high temporal resolution signals. To our knowledge, we are the first to study the problem of building pretrained channel aggregation models on top of pre-existing temporal embeddings trained across neural datasets with variable channel layouts, allowing for modeling of high quality neural data.

### Modeling across variable input channels

Modeling spatial representations on top of temporal embeddings has been found to be beneficial for decoding (Faezi et al., 2021; Le & Shlizerman, 2022; Azabou et al., 2024), but prior works use supervised labels, so do not leverage large amounts of unannotated data. The brain-computer-interface field has studied how to align latent spaces (Pandarinath et al., 2018; Karpowicz et al., 2022; Degenhart et al., 2020; Jude et al.; Ma et al., 2023) which either still requires creating an alignment matrix to learn across datasets or only provides post-training alignment mechanisms rather than learning across datasets. Other approaches impute missing channels or learn latent spaces robust to missing channels (Talukder et al., 2022; Zhang et al., 2021; Chau et al., 2024), but these are more suited for the occasional missing channel rather than largely varying sensor layouts. We directly learn spatial-level representations using self-supervised learning across datasets to leverage massive amounts of unannotated intracranial data.

## 3 Population Transformer Approach

**Figure 1:**
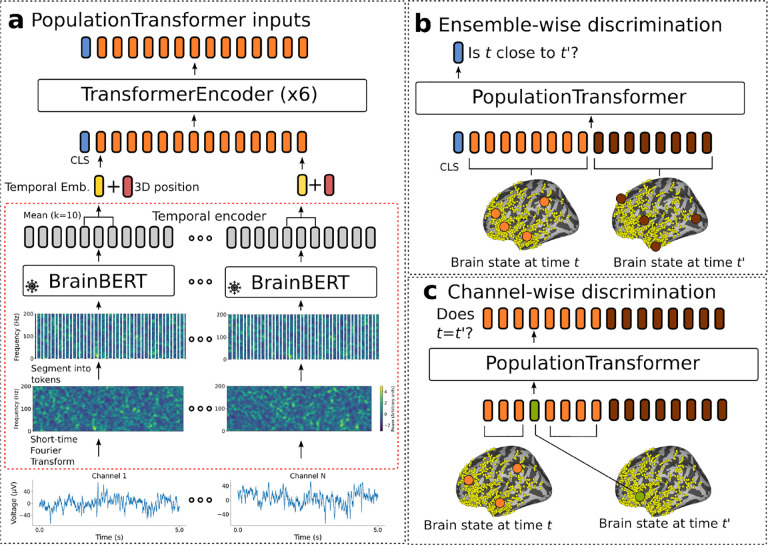
Schematic of our approach. The inputs to our model (a) are the combined neural activities from a collection of intracranial electrodes in a given time interval. These are passed to a frozen temporal embedding model (dotted red outline: BrainBERT shown), which produces a set of time-contextual embedding vectors (yellow). The 3D position of each electrode (red) is added to these vectors to produce the model inputs (orange). The PopT produces space-contextual embeddings for each electrode and a [CLS] token (blue), which can be fine-tuned for downstream tasks. During pretraining, the PopT is trained on two objectives simultaneously. In the first, (b) the PopT determines whether two different sets of electrodes (orange vs brown) represent consecutive or non-consecutive times. In the second objective, (c) the PopT must determine whether an input channel has been replaced with activity at a random other time that is inconsistent with the majority of inputs.

Figure 1 overviews our Population Transformer (PopT) approach. The key ideas are: (1) to learn a generic representation of neural recordings that can handle arbitrary electrode configurations; and (2) to employ a modular system design that uses a transformer architecture to aggregate information from existing per-channel temporal embeddings. To do so, we employ a self-supervised pretraining approach to learn ensemble and channel level representations. Afterwards, one can fine-tune PopT on downstream decoding tasks. In addition to offering strong decoding results, including generalization to new subjects with different electrode configurations than training subjects (see Section 5), the modular system design is computationally lightweight (see Appendix B), can benefit from improved temporal representations, and is more readily interpretable (see Section 6).

### Architecture

A schematic of our Population Transformer (PopT) approach is shown in Figure 1. We adapt a transformer backbone due to its ability to accommodate variable channel configurations. Consider a given subject with *N* channels indexed by *C* = {1, …*, N*_*c*_}, and an arbitrary subset of channels *S* ⊆ *C*. Activity from channel *i* at time *t* can be denoted by $x_{i}^{t}$. The PopT takes as input an interval of brain activity $X^{t}=\left\{x_{i}^{t}|i\in S\right\}$ from a given time *t* and a special [CLS] token. Per channel, each interval of brain activity is passed through a temporal embedding model *B*, in the figure’s case BrainBERT, to obtain a representation of each channel’s temporal context.

To allow the model to learn a common brain state representation across layouts, each channel’s embedding is augmented with its 3D position, so that the final input to the PopT is $X_{B}^{t}=\left\{B\left(x_{i}^{t}\right)+\text{pos}(i)+𝒩(0,\sigma )|x_{i}^{t}\in X^{t}\right\}$. Spatial location is given by the electrode’s Left, Posterior, and Inferior coordinates for iEEG electrodes (Wideman, 2024), and XYZ positions for EEG electrodes. We add Gaussian fuzzing to each coordinate location to prevent overfitting to a particular set of coordinates. Each coordinate is then encoded using sinusoidal position encoding (Vaswani et al., 2017). Membership in a particular ensemble (see below: ensemble-wise loss) is also encoded. The four encodings are concatenated together to form the position embedding *pos*(*i*) = [*e*_left_; *e*_post_.; *e*_inf_; *e*_ensemble_].

The core of PopT consists of a transformer encoder stack (see Appendix A: Architectures). The output of the PopT are spatial-contextual embeddings of the channels *Y* = {*y*_*i*_} as well as an embedding of the CLS token *y*_*cls*_. During pretraining, the PopulationTransformer additionally is equipped with a linear layer head for the [CLS] token output and separate linear layer heads for all other individual token outputs. These produce the scalars ${\tilde{y}}_{cls}$ and ${\tilde{y}}_{i}$ respectively, which are used in the pretraining objective (see Figure 1b and c).

### Self-supervised loss

Our loss function has two discriminative components: (1) *ensemble-wise* —the model determines if activities from two channel ensembles occurred consecutively, requiring an effective brain state representation at the ensemble-level, (2) *channel-wise* — the model identifies outlier channels that have been swapped with a different timepoint’s activity, requiring sensitivity to surrounding channel context.

A key aspect of our method is the fact that our objective is discriminative, rather than reconstructive, as is often the case in self-supervision (Liu et al., 2021; Wang et al., 2022). We found this to be necessary, because in practice, the temporal embeddings often have low effective dimension (see Wang et al. (2022)), and reconstruction rewards the model for overfitting to “filler” dimensions in the feature vector (see Section 5).

### Pretraining

In *ensemble-wise discrimination* (fig. 1b), two different subsets of channels *S*_*A*_*, S*_*B*_ ⊂ *C* are chosen with the condition that they be disjoint *S*_*A*_ ∩ *S*_*B*_ = ∅. During pretraining, the model receives the activities from these channels at separate times $X_{A}^{t}=\left\{x_{i}^{t}|i\in S_{A}\right\}$ and $X_{B}^{t^{\prime }}=\{x_{i}^{t^{\prime }}|i\in S_{B}\}$ . The objective of the task is then to determine whether these states $X_{A}^{t}$ and $X_{B}^{t^{\prime }}$ have occurred consecutively in time or are separated by some further, randomly selected interval. Given the output of the classification head, the loss function ℒ_*N*_ is again the binary cross entropy. We also vary the number of input channels during sampling to ensure the model handles ensembles of different sizes.

Additionally, we select disjoint subsets for ensemble-wise discrimination to prevent the model from solving tasks through trivial copying.

In *channel-wise discrimination* (fig. 1c), the model must determine whether a channel’s activity has been swapped with activity from a random time. Precisely, activity from each channel *i* is drawn from a time *t*_*i*_. All channels are drawn from the same time *t*_*i*_ = *T*, and then 10% of the channels are randomly selected to have their activity replaced with activity from the same channel, but taken from a random point in time *t*_*i*_ ≠ *T*. Then, given the token outputs of PopT, the channel-wise loss function ℒ_*C*_ is the binary cross entropy. Then, our complete objective function is ℒ = ℒ_*N*_ + ℒ_*C*_.

### Fine-tuning

During fine-tuning, the [CLS] intermediate representation, ${\tilde{y}}_{cls}$ of the pretrained PopT is passed through a single layer linear neural network to produce a scalar ${\hat{y}}_{cls}$; this forms the input to the binary cross entropy loss for our decoding tasks (see Section 4).

## 4 EXPERIMENT SETUP

### Data

We use two types of neural time-series data: intracranial and scalp electroencepholography (iEEG and EEG). Whereas iEEG probes are surgically implanted within the 3D brain volume, EEG electrodes lie on the scalp. These cover two resolution extremes of neural time-series data modalities.

*iEEG:* We use the publicly available subject data from Wang et al. (2022). Data was collected from 10 subjects (total 1,688 electrodes, with a mean of 167 electrodes per subject) who watched 26 movies while intracranial probes recorded their brain activity. Of the sessions, 19 are used for pretraining, and 7 of the sessions are held-out for evaluation.
*EEG:* We use the Temple University Hospital EEG and Abnormal datasets (TUEG and TUAB (Obeid & Picone, 2016)) for pretraining and task data respectively. We remove all task subjects from the pretraining set and follow the data preprocessing practices in Yang et al. (2024); Jiang et al. (2024).

### Decoding Tasks

We evaluate on 5 different classification tasks: 4 auditory-linguistic tasks used in the evaluation of Wang et al. (2022) and 1 widely evaluated seizure detection task from Obeid & Picone (2016). Of the auditory-linguistic tasks, two of the tasks are audio focused: determining whether a word is spoken with a high or low pitch and determining whether a word is spoken loudly or softly. And two of the tasks have a more linguistic focus: determining whether the beginning of a sentence is occurring or determining whether any speech at all is occurring. The EEG TUAB seizure detection task is a binary classification of pathological or normal EEG recording.

To test scaling with arbitrary ensemble sizes, we select subsets of electrodes based on their individual linear decodability, with the smallest subsets containing the electrodes with highest decodability.

### Baselines

For controlled baselines, we concatenate the single-channel temporal embeddings and train a linear (Linear) or non-linear (Deep NN) aggregator on the decoding task. These enable us to directly assess how much PopT improves upon existing aggregation approaches (Ghosal & Abbasi-Asl, 2021). These approaches cannot be pretrained across subjects due to the changing meaning and quantity of inputs. To test the effectiveness of pretraining, we also compare against a non-pretrained PopT.

### Methods compared

For the iEEG experiments, we also compare against Brant (Zhang et al., 2024), which is an end-to-end iEEG trained encoder. We take the fully pretrained Brant model, and fine-tune on our iEEG tasks combining channels with linear aggregation. For the EEG experiments, we compare against BIOT (Yang et al., 2024) and LaBraM (Jiang et al., 2024). Both BIOT and LaBraM use discrete channel ID as positional encodings, which only allows them to be used for layouts with discete channel locations such as EEG. They also train end-to-end in contrast to our modular approach.

## 5 Results

### Decoding performance

**Figure 2:**
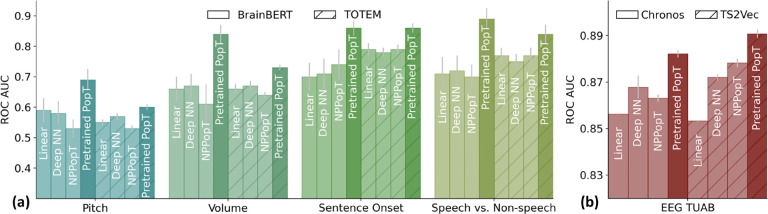
Compared to common aggregation approaches, pretrained PopT consistently yields better downstream decoding across tasks, data modalities, and temporal embedding types. NPopT = Non-pretrained PopT. (a) performance on four audio-linguistic iEEG tasks with 90 electrodes. Grey bars denote standard error across subjects. (b) performance on a seizure detection EEG task with 21 electrodes. Grey bars denote standard deviation across 5 random seeds.

**Figure 3:**
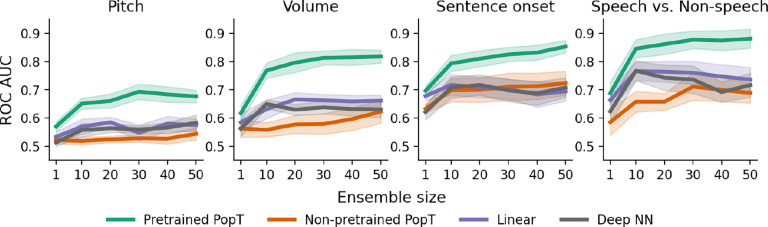
Pretrained PopT downstream performance scales better with ensemble size. Increasing channel ensemble size from 1 to 50 (x-axis), we see pretrained PopT (green) decoding performance (y-axis) not only beat non-pretrained approaches (orange, purple, grey), but also continually improve more with increasing channel count. Shaded bands show the standard error across subjects.

We find that using a pretrained PopT significantly benefits downstream decoding compared to baseline channel aggregation techniques across tasks, data modalities, and temporal encoding models (Tables 1 and 2 and fig. 2). The pretrained PopT scales well with increasing ensemble sizes (Figure 3a), a challenging task for the baseline aggregation approaches due to limited downstream task data and increasing input size. To test our method’s ability to handle multiple types of channel encodings, we applied our framework to 4 different channel encoders: (1) an iEEG-specific temporal encoder (BrainBERT (Wang et al., 2022)), (2) a general tokenization-based time-series encoder (TOTEM (Talukder et al., 2024)), (3) a pretrained general time-series encoder (Chronos (Ansari et al., 2024)), and a general convolution-based time-series encoder (TS2Vec (Yue et al., 2022)). We see significant improvements in performance with the pretrained PopT in all cases when comparing with baseline aggregation approaches (Figure 2).

We also find that PopT can achieve competitive performance against pretrained end-to-end models, such as Brant (Zhang et al., 2024) for iEEG, and BIOT (Yang et al., 2024) and LaBraM (Jiang et al., 2024) for EEG (Tables 1 and 2). For instance, PopT outperforms Brant (Zhang et al., 2024) in decoding iEEG data with our pretrained PopT + BrainBERT combination, likely due to PopT’s ability to leverage spatial relationships. Whereas Brant leaves the channel aggregation problem open. PopT is competitive with recent end-to-end trained EEG models (Yang et al., 2024; Jiang et al., 2024) at the EEG TUAB seizure detection task. This is impressive, since models such as LaBraM were specifically developed for this application, whereas PopT was trained on top of generic time-series embeddings. We find that PopT can offer an efficient and competitive alternative to large end-to-end models for these decoding tasks, due to the effectiveness of our pretraining task for learning spatial and functional relationships between channel input embeddings.

To verify that the weights of the pretrained PopT capture neural processing well even without finetuning, we also train a linear-encoder on top of the frozen PopT [CLS] token and find the same trends (Figure 11. This point in particular is important in building confidence in the results of our interpretability studies (see Section 6), in which we use the frozen pretrained weights to analyze connectivity. Finally, for the remaining analyses described below, we use a PopT with BrainBERT inputs.

### Sample and compute efficiency

**Figure 4:**
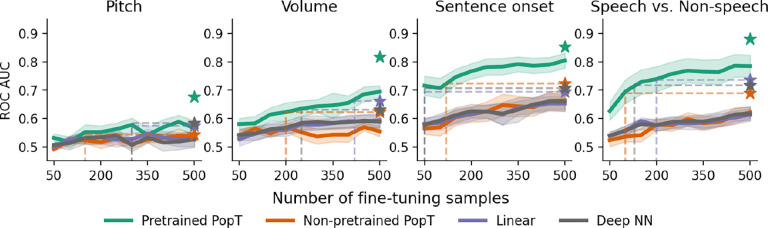
Pretrained PopT is more sample efficient when fine-tuning. Varying the number of samples available to each model at train time (x-axis), we see that the pretrained PopT is highly sample efficient, requiring only a fraction of samples (fewer than 500 samples out of 5–10k of the full dataset) to reach the full performance level of baseline aggregation approaches (dashed lines). Bands show standard error across test subjects. Stars indicate performance with full fine-tuning dataset.

**Figure 5:**
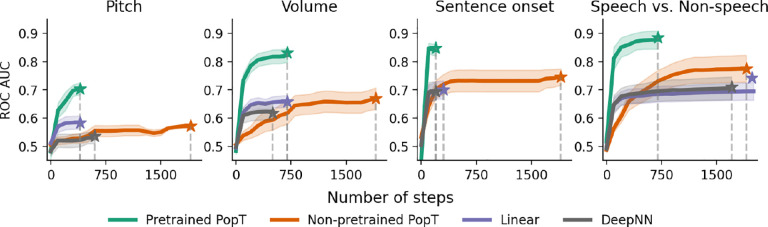
Pretrained PopT is consistently compute efficient when fine-tuning. Number of steps required for each model to reach final performance during fine-tuning (dashed lines). The pretrained PopT consistently requires fewer than 750 steps (each step is an update on a batch size of 256) to converge, in contrast to the 2k steps required for the non-pretrained PopT. Linear and Deep NN aggregation can be similarly compute efficient, but occasionally require more training steps depending on task. Bands show standard error across subjects. Stars indicate fully trained performance.

Our PopT learns spatial relationships between channels, in a way that makes downstream supervised learning more data and compute efficient (see Figure 4 and Figure 5). Compared to the non-pretrained baseline models, fine-tuning the pretrained PopT can achieve the same decoding performance as other aggregation techniques with an order of magnitude fewer samples. The pretrained PopT surpasses the performance achieved by all other aggregation techniques by 500 samples out of the full dataset (roughly 5–10k examples depending on subject and task) Figure 4. The pretrained PopT also converges at a low number of steps, competitive with Linear and Deep NN compute efficiency. This greatly contrasts with the non-pretrained PopT and occasionally the Linear and Deep NN baselines, which may require 2k or more steps (Figure 5).

### Generalizability

**Figure 6:**
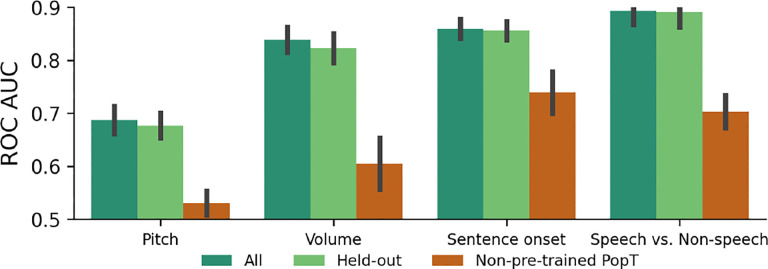
Gains in decoding performance are available to new subjects. A minimal decrease in downstream decoding performance is found if the subject is held-out from pretraining (Held-out vs All). This is in stark contrast to the achievable downstream performance with a non-pretrained PopT (Non-pretrained PopT).

To test if our pretrained weights will be useful for subjects not seen during training, we conduct a hold-one-out analysis. We pretrain a model using all subjects except for one, and then fine-tune and evaluate on the model downstream. We find that missing a subject from pretraining does not significantly affect the downstream results (see Figure 6). This raises our confidence that the pretrained weights will be useful for unseen subjects and for researchers using new data.

### Scaling with number of pretraining subjects

**Figure 7:**
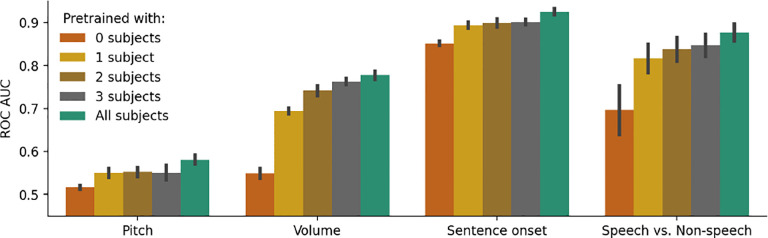
Pretraining with more subjects leads to better downstream performance. We pretrain PopT with different number of subjects (colors) and test on our decoding tasks (x-axis). Bars indicate mean and standard error of performance across channel ensembles 5–30 on a held out test subject. Pretraining with one subject gives a considerable benefit compared to no pretraining (red to yellow), but the addition of more subjects to pretraining consistently improves performance (yellow → green).

To investigate the effect of scaling pretraining data on our model, we pretrain additional versions of PopT using only 1, 2, or 3 subjects. We find a consistent improvement in downstream decoding when we increase the number of pretraining subjects available across all our downstream decoding tasks Figure 7. A significant improvement is found with just 1 pretraining subject already, potentially due to adaption to the temporal embeddings used. The decoding performance using all our pretraining data is significantly higher in most decoding tasks than with just 1 or 2 subjects in the pretraining data, suggesting the potential for our framework to continue scaling with more subjects.

### Ablation of loss components and position information

An ablation study confirms that both the ensemble-wise and channel-wise component of the pretraining objective contribute to the downstream performance (Table 3). Furthermore, including the 3D position information for each channel is critical for decoding. These findings also hold when the PopT is kept frozen during fine-tuning (see Appendix H: Frozen ablation). Additionally, we find that the discriminative nature of our loss is necessary for decoding. Attempting to add an L1 reconstruction term to our pretraining objective results in poorer performance, perhaps because the model learns to overfit on low-entropy features in the embedding. Our discriminative loss requires the model to understand the embeddings in terms of how they can be distinguished from one another, which leads the model to extract more informative representations.

## 6 Interpreting Learned Weights

Finally, we conduct two interpretability studies of the Population Transformer’s learned weights.

### Connectivity

**Figure 8:**
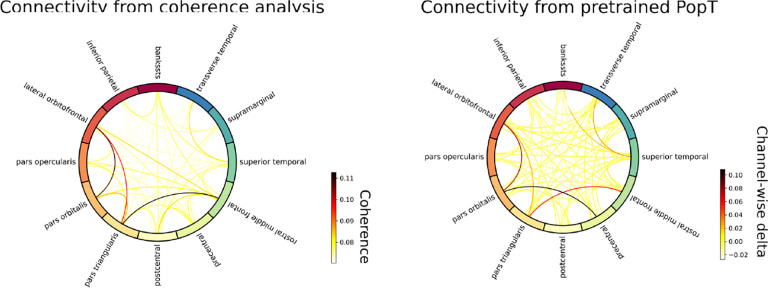
Probing the pretrained model for inter-channel connectivity Traditionally, connectivity analysis between regions is done by computing the coherence between electrode activity (left). We propose an alternative analysis purely based on the contextual sensitivity learned during pretraining. Briefly, we select an electrode, mask out its activity, and then measure the degradation in the channel-wise objective function for the remaining electrodes. Plotting the values of this delta (right) recovers the main points of connectivity. Plots for all test subjects can be seen in Appendix F: Connectivity.

Traditional neuroscience analyses typically use cross-correlation as a measure of region connectivity (Wang et al., 2021). Our PopT allows for an alternative method of determining connectivity, based on the degree to which channels are sensitive to each other’s context. In this method, each channel is masked in turn, and then model performance on the pretraining channel-wise objective for the remaining unmasked channels is measured. We use the degradation in performance as a measure of connectivity. We can construct plots as in Figure 8, that recapitulate the strongest connectivity of the cross-correlation maps. Note that while some approaches for modelling brain activity explicitly build this into their architecture (Cai et al., 2023), we recover these connections purely as a result of our self-supervised learning. Additional method details available in Appendix E.

### Candidate functional brain regions from attention weights

**Figure 9:**
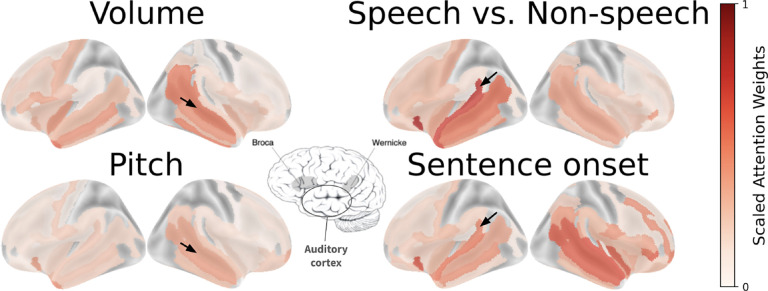
Attention weights from a fine-tuned PopT identify candidate functional brain regions Candidate functional maps can be read from attention weights of a PopT fine-tuned on our decoding tasks. For the Volume and Pitch tasks, note the weight placed on the primary auditory cortex (black arrows), but not in Wernicke’s area. For the Speech vs Non-speech and Sentence onset tasks, note the weight placed on regions near Wernicke’s area (black arrows). Center brain figure highlight regions related to auditory-linguistic processing (aph, 2017)).

**Figure 11:**
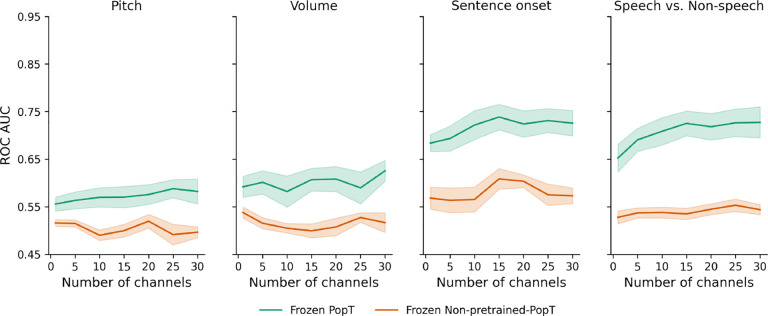
Pretraining is critical to frozen PopT performance that scales with the number of channels. As in Figure 3, we see that pretraining results in better downstream decoding and better scaling with the number of added channels. However, unlike in Figure 3, the PopT weights are frozen during fine-tuning, and only the linear classification head is updated. Bands show standard error across subjects. Results are shown for a frozen PopT with BrainBERT inputs.

After fine-tuning our weights on a decoding task, we can examine the attention weights of the [CLS] output for candidate functional brain regions. We obtain a normalized Scaled Attention Weight metric across all subjects to analyze candidate functional brain regions across sparsely sampled subject datasets Figure 9. The Scaled Attention Weight is computed from raw attention weights at the [CLS] token passed through the attention rollout algorithm (Abnar & Zuidema, 2020). The resulting weights from each channel are then grouped by brain region according to the Destrieux atlas (Destrieux et al., 2010). A full description of the method is available in Appendix E.

The resulting weights reveal expected functional brain regions related to the tasks decoded Figure 9, with low-level auditory tasks highlighting primary auditory cortex and higher-level language distinction tasks highlighting language-specific areas. Given the massive pretraining PopT undergoes, these scaled attention weights provide a valuable new tool for discovering candidate functional regions.

## 7 Discussion

We presented a self-supervised scheme for learning effective joint representations of neural activity from temporal embeddings. Our approach improves decoding and reduces the samples required to learn downstream tasks, which is especially critical for neural data modalities given patient constraints. A key aspect of our approach is the fact that we focus on spatial aggregation of existing channel embeddings, rather than training a large end-to-end model. By decoupling temporal and spatial feature extraction, we are able to leverage existing temporal embeddings to learn spatiotemporal representations efficiently and with a smaller number of parameters. This makes our model available for use in low compute-resource settings. Furthermore, this separation of considerations opens up the possibility for future independent improvement in temporal modeling, whether that be from a domain specific model or a more general time-series encoder. The generality of this approach allowed us to train on two very different neural modalities: scalp EEG and invasive iEEG. Our success in these domains suggest that this approach could even be extended to settings outside of neuroscience that also contend with sparsely and variably distributed time-series data channels, as is often the case with geophysical or climate data.

### Limitations and Future Work

We propose an effective strategy for aggregating signals with meaningful spatial coordinates, but it remains to be seen how we can generalize this further to aggregations without such coordinates. Individual brains are also highly variable, so improving this positional encoding scheme could provide an even higher resolution latent space of each individual’s brain. Future work could experiment with automatic functional identification for each channel, such as that explored in neural spiking data (Azabou et al., 2024), but it is currently unclear how to do so with neural recordings that have lower SNR.

## 8 Conclusion

We introduced a pretraining method for learning representations of arbitrary ensembles of intracranial electrodes. We showed that our pretraining produced considerable improvements in downstream decoding and efficiency, that would not have been possible without the knowledge of spatial relationships learned during the self-supervised pretraining stage. These benefits were found across data modalities, decoding tasks, and temporal encoders used, speaking to the generality of our approach. We further showed that this scheme produces interpretable weights from which connectivity maps and candidate functional brain regions can be read. Finally, we release the pretrained weights for our PopT with BrainBERT inputs as well as our code for plug-and-play pretraining with any temporal embedding.

## Figures and Tables

**Table 1:** Pretraining PopT is critical to downstream decoding performance (iEEG data). We test on a variety of audio-linguistic decoding tasks (see Section 4) with 90 channels as input. The temporal encoder used for aggregation in sections 1 and 2 are denoted in the section header. We also evaluate against an end-to-end pretrained iEEG model in section 3. Shown are the ROC-AUC mean and standard error across subjects. Best per section are bolded.

Model	Pitch	Volume	Sent. Onset	Speech/Non-speech
BrainBERT:				
Linear Agg.	0.59 ± 0.08	0.66 ± 0.08	0.70 ± 0.09	0.71 ± 0.11
Deep NN Agg.	0.58 ± 0.08	0.67 ± 0.08	0.71 ± 0.10	0.72 ± 0.10
Non-pretrained PopT	0.53 ± 0.06	0.61 ± 0.13	0.74 ± 0.10	0.70 ± 0.08
Pretrained PopT	**0**.**69** ± **0**.**07**	**0**.**84** ± **0**.**06**	**0**.**86** ± **0**.**05**	**0**.**89** ± **0**.**07**
TOTEM:				
Linear Agg.	0.55 ± 0.02	0.66 ± 0.03	0.79 ± 0.04	0.77 ± 0.05
Deep NN Agg.	0.57 ± 0.02	0.67 ± 0.03	0.78 ± 0.03	0.75 ± 0.05
Non-pretrained PopT	0.53 ± 0.02	0.64 ± 0.02	0.79 ± 0.03	0.77 ± 0.05
Pretrained PopT	**0**.**60** ± **0**.**02**	**0**.**73** ± **0**.**02**	**0**.**86** ± **0**.**03**	**0**.**84** ± **0**.**06**
End-to-end:				
Brant (Zhang et al., 2024)	**0**.**61** ± **0**.**03**	**0**.**74** ± **0**.**03**	**0**.**80** ± **0**.**04**	**0**.**80** ± **0**.**03**

**Table 2:** Pretraining PopT is critical to downstream decoding performance (EEG data). We test on a seizure detection task (TUAB in Obeid & Picone (2016)) with 21 channels as input. The temporal encoder used for aggregation in sections 1 and 2 are denoted in the section header. We also evaluate against end-to-end pretrained EEG models in section 3 (values from the original works). Shown are the ROC-AUC mean and stdev across 5 random seeds. Best per section are bolded.

Model	Balanced Accuracy	ROC AUC
Chronos		
Linear Agg.	0.7754 ± 0.0008	0.8563 ± 0.0003
Deep NN Agg.	0.7881 ± 0.0057	0.8678 ± 0.0049
Non-pretrained PopT	0.7763 ± 0.0047	0.8631 ± 0.0016
Pretrained PopT	**0**.**7976** ± **0**.**0022**	**0**.**8821** ± **0**.**0016**
TS2Vec		
Linear Agg.	0.7649 ± 0.0005	0.8533 ± 0.0003
Deep NN Agg.	0.7853 ± 0.0021	0.8721 ± 0.0015
Non-pretrained PopT	0.7896 ± 0.0037	0.8782 ± 0.0018
Pretrained PopT	**0**.**8063** ± **0**.**0010**	**0**.**8907** ± **0**.**0019**
End-to-end:		
BIOT (Yang et al., 2024)	0.7959 ± 0.0057	0.8815 ± 0.0043
LaBraM (Jiang et al., 2024)	**0**.**8258** ± **0**.**0011**	**0**.**9162** ± **0**.**0016**

**Table 3:** PopT ablation study. We individually ablate our losses and positional encodings during pretraining then decode on the resulting models. Shown are ROC-AUC mean and standard error across subjects evaluated at 90 electrodes. The best performing model across all decoding tasks uses all of our proposed components, showing that they are all necessary. Removing our positional encoding during pretraining and fine-tuning drops the performance the most, indicating that position encoding is highly important for achieving good decoding. Additionally, we attempt adding or only using a reconstruction component to the loss as a regularizing term, but find that this leads to poorer performance (last two rows).

	Pitch	Volume	Sent. Onset	Speech/Non-speech
PopT	**0**.**69** ± **0**.**07**	**0**.**84** ± **0**.**06**	**0**.**86** ± **0**.**05**	**0**.**89** ± **0**.**07**
PopT w/o group-wise loss	0.66 ± 0.07	0.83 ± 0.06	0.84 ± 0.04	0.88 ± 0.08
PopT w/o channel-wise loss	0.67 ± 0.06	0.81 ± 0.08	0.84 ± 0.06	0.87 ± 0.09
PopT w/o position encoding	0.59 ± 0.07	0.67 ± 0.10	0.75 ± 0.08	0.79 ± 0.08
PopT with reconstruction loss	0.60 ± 0.11	0.73 ± 0.11	0.81 ± 0.05	0.83 ± 0.09
PopT with L1 reconstruction only	0.56 ± 0.04	0.65 ± 0.08	0.73 ± 0.10	0.74 ± 0.10

**Figure 10:**
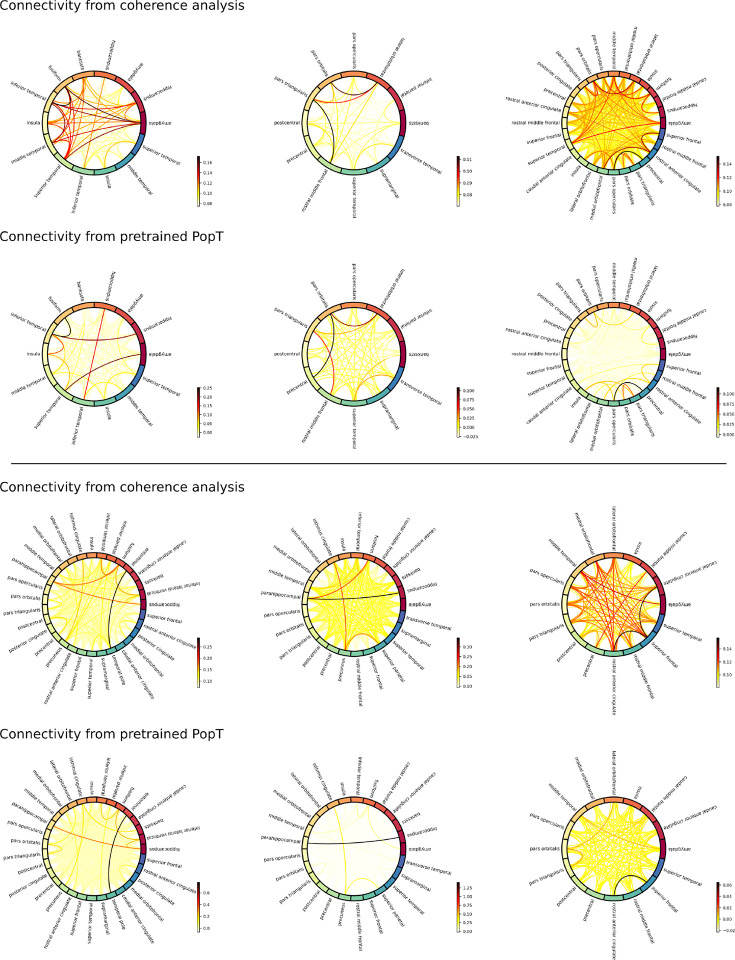
Full connectivity for our 7 test subjects. We compare between traditional connectivity analaysis performed via coherence (top row in each section) and the analysis based on our PopT pretrained weights (bottom row in each section). We note that our analysis usually recovers the strongest points of connectivity fromt the traditional analysis. Coherence was computed using scikit-learn’s (Pedregosa et al., 2011) signal.coherence.

## A Architectures and training

### Pretrained PopT

The core Population Transformer consists of a transformer encoder stack with 6 layers, 8 heads. All layers (*N* = 6) in the encoder stack are set with the following parameters: *d*_*h*_ = 512, *H* = 8, and *p*_dropout_ = 0.1. We pretrain the PopT model with the LAMB optimizer (You et al., 2019) (*lr* = 1*e* − 4), with a batch size of *n*_batch_ = 256, and train/val/test split of 0.98, 0.01, 0.01 of the data. We pretrain for 500,000 steps, and record the validation performance every 1,000 steps. Downstream evaluation takes place on the weights with the best validation performance. We use the intermediate representation at the [CLS] token *d*_*h*_ = 512 and put a linear layer that outputs to *d*_*out*_ = 1 for fine-tuning on downstream tasks. These parameters for pretraining were the same for any PopT that needed to be pretrained (hold-one-out subject, subject subsets, ablation studies).

### Non-pretrained PopT

The architecture for the non-pretrained PopT is the same as the pretrained PopT (above). However, no pretraining is done, and the weights are randomly initialized with the default initializations.

### Linear

The linear baseline consists of a single linear layer that outputs to *d*_*out*_ = 1. The inputs are flattened and concatenated BrainBERT embeddings *d*_*emb*_ = 756, TOTEM embeddings *d*_*emb*_ = 64, Chronos embeddings *d*_*emb*_ = 512, or TS2Vec embeddings *d*_*emb*_ = 320 from a subset of channels *S* ⊂ *C*. Thus, the full input dimension is *d*_*input*_ = *d*_*emb*_ * |*S*|.

### Deep NN

The inputs are the same as above, but the decoding network now consists of 5 stacked linear layers, each with *d*_*h*_ = 512 and a GeLU activation.

### Downstream Training

For both PopT models, we train with these parameters: AdamW optimizer (Loshchilov & Hutter, 2017), *lr* = 5*e*^−4^ where transformer weights are scaled down by a factor of 10 (*lr*_*t*_ = 5*e*^−5^), *n*_*batch*_ = 256, a Ramp Up scheduler (ildoonet, 2024) with warmup 0.025 and Step LR gamma 0.99, reducing 100 times within the 2000 total steps that we train for. For Linear and DeepNN models, we train with these parameters: AdamW optimizer (Loshchilov & Hutter, 2017), *lr* = 5*e*^−4^, *n*_*batch*_ = 256, a Ramp Up scheduler (ildoonet, 2024) with warmup 0.025 and Step LR gamma 0.95, reducing 25 times within the 17,000 total steps we train for. For all downstream decoding, we use a fixed train/val/test split of 0.8, 0.1, 0.1 of the data.

### Compute Resources

To run all our experiments (data processing, pretraining, evaluations, interpretability), one only needs 1 NVIDIA Titan RTXs (24GB GPU Ram). Pretraining PopT takes 2 days on 1 GPU. Our downstream evaluations take a few minutes to run each. For the purposes of data processing and gathering all the results in the paper, we parallelized the experiments on 8 GPUs.

## B Model and Compute Requirements

**Table 4:** Parameter counts. Since PopT takes existing temporal embeddings as input, the number of parameters that must be trained is an order of magnitude less than recent end-to-end approaches.

	e5	e50	e90
PopT		20M	
Deep NN	3M	20M	36M
Linear	3.8k	38k	69k
Brant (Zhang et al., 2024)		500M	
LaBraM (Jiang et al., 2024)		350M	

**Table 5:** Pretraining compute requirements Based on published train times (none were given for LaBraM) it is evident that PopT has smaller hardware and shorter training time requirements.

	GPU count	GPU type	Time to train	TFLOPS
PopT	1	NVIDIA TITAN RTX (24GB)	2 days	2.1M
Brant (Zhang et al., 2024)	4	NVIDIA Tesla A100 (80G)	2.8 days	18.8M
LaBraM (Jiang et al., 2024)	8	NVIDIA Tesla A800 (40G)	–	–

## C Decoding tasks

We follow the same task specification as in Wang et al. (2022), with the modification that the pitch and volume examples are determined by percentile (see below) rather than standard deviation in order to obtain balanced classes.

### Pitch

The PopT receives an interval of activity and must determine if it corresponds with a high or low pitch word being spoken. For the duration of a given word, pitch was extracted using Librosa’s piptrack function over a Mel-spectrogram (sampling rate 48,000 Hz, FFT window length of 2048, hop length of 512, and 128 mel filters). For this task, for a given session, positive examples consist of words in the top-quartile of mean pitch and negative examples are the words in the bottom quartiles.

### Volume

The volume of a given word was computed as the average intensity of root-mean-square (RMS) (rms function, frame and hop lengths 2048 and 512 respectively). As before, positive examples are the words in the top-quartile of volume and negative examples are those in the bottom quartiles.

### Sentence onset

Negative examples are intervals of activity from 1s periods during which no speech is occurring in the movie. Positive examples are intervals of brain activity that correspond with hearing the first word of a sentence.

### Speech vs. Non-speech

Negative examples are as before. Positive examples are intervals of brain activity that correspond with dialogue being spoken in the stimuli movie.

## D Data

**Table 6:** Subject statistics Subjects used in PopT training, and held-out downstream evaluation. Table taken from Wang et al. (2022). The second column shows the number of uncorrupted, electrodes that can be Laplacian re-referenced. The average amount of recording data per subject is 4.3 (hrs).

Subj.	Age (yrs.)	# Electrodes	Movie	Recording time (hrs)	Held-out
	19	91	Thor: Ragnarok	1.83	
1			Fantastic Mr. Fox	1.75	
			The Martian	0.5	x
	12	100	Venom	2.42	
			Spider-Man: Homecoming	2.42	
			Guardians of the Galaxy	2.5	
2			Guardians of the Galaxy 2	3	
			Avengers: Infinity War	4.33	
			Black Panther	1.75	
			Aquaman	3.42	x
	18	91	Cars 2	1.92	x
3			Lord of the Rings 1	2.67	
			Lord of the Rings 2 (extended edition)	3.92	
4	9	135	Megamind	2.58	
			Toy Story	1.33	
			Coraline	1.83	x
	11	205	Cars 2	1.75	x
5			Megamind	1.77	
	12	152	Incredibles	1.15	
6			Shrek3	1.68	x
			Megamind	2.43	
7	6	109	Fantastic Mr. Fox	1.5	
8	4.5	72	Sesame Street Episode	1.28	
9	16	102	Ant Man	2.28	
10	12	173	Cars 2	1.58	x
			Spider-Man: Far from Home	2.17	

## E Interpretation Methods

### Connectivity analysis

We start with a pretrained PopT. To test a particular channel’s contribution to connectivity, we mask it with all zeros. Then, we consider the remaining unmasked channels and ask, how much increase do we see in the pretraining channel-wise loss? Recall that this objective is to determine whether or not a channel has had its inputs swapped with random activity. If the increase in loss is large, then we infer that the masked channel provided important context for this task. Using this delta as a measure for connectivity, we then average across the Desikan-Killiany atlas regions (Alexander et al., 2019) and produce a plot using mne-connectivity (Gramfort et al., 2013).

### Scaled Attention Weight

First, we obtain an attention weight matrix across all trials which includes weights between all tokens. Then, we perform attention rollout (Abnar & Zuidema, 2020) across layers to obtain the contributions of each input channel by the last layer. We take the resulting last layer of rollout weights for all channels, where the target is the [CLS] token, normalize within subject, and scale by ROC AUC to obtain the Scaled Attention Weight per channel. Finally, we plot the 0.75 percentile weight per region, as mapped by the Destrieux atlas (Destrieux et al., 2010) using Nilearn (contributors).

## H Frozen ablation

**Table 7:** An ablation study of the components of our approach for the frozen PopT. During pretraining, we alternate using either only the CLS or token contrastive component of the loss. We fine-tune these weights on all subjects. We find that both components contribute to the full model’s performance.

	Sentence onset	Speech/Non-speech	Pitch	Volume
Frozen PopT	**0**.**73** ± **0**.**06**	**0**.**72** ± **0**.**08**	0.59 ± 0.06	**0**.**63** ± **0**.**07**
w/o cls	0.67 ± 0.08	0.68 ± 0.07	0.58 ± 0.04	0.60 ± 0.07
w/o replace loss	0.69 ± 0.07	0.69 ± 0.09	**0**.**59** ± **0**.**06**	0.62 ± 0.06
w/o position encoding	0.70 ± 0.07	0.69 ± 0.07	0.56 ± 0.08	0.61 ± 0.06
